# Waking Up Younger Together: Better Sleep Quality is Linked to a Younger Subjective Age in Older Couples

**DOI:** 10.1093/geroni/igaf122.3106

**Published:** 2025-12-31

**Authors:** Sierra Birthelmer, Juhyeong Lee, Elizabeth Zambrano Garza, Yoonseok Choi, Klaus Rothermund, Carsten Wrosch, Denis Gerstorf, Christiane Hoppmann

**Affiliations:** The University of British Columbia, Vancouver, British Columbia, Canada; The University of British Columbia, Vancouver, British Columbia, Canada; The University of British Columbia, Vancouver, British Columbia, Canada; Simon Fraser University, Vancouver, British Columbia, Canada; Friedrich-Schiller University Jena, Institute of Psychology, Jena, Thuringen, Germany; Concordia University, Montreal, Quebec, Canada; Humboldt University Berlin, Humboldt University Berlin, Berlin, Germany; UBC, Vancouver, British Columbia, Canada

## Abstract

The Contextual Model of Subjective Age proposes that daily experiences, including health and recovery, influence the salience of one’s age leading to within-individual variability in subjective age. Daily sleep quality may be one indicator shaping subjective age. Since both sleep quality and subjective age can be shaped by social partners, this study extended past work by examining daily life fluctuations in sleep quality and subjective age among Canadian older couples. We analyzed data from 67 older couples living in Canada (Age: M = 68.4 years, SD = 6.3; 49% female) who provided morning ratings of sleep quality and subjective age over ten consecutive days. We pre-registered the study. After accounting for covariates, findings using the Actor-Partner Interdependence Model indicate that, as hypothesized, increases in sleep quality in both actors and partners were associated with decreases in subjective age. This suggests that when older adults and their partners experience better sleep quality, they report a younger subjective age upon waking, relative to days following poor sleep quality. These findings will be discussed in the context of daily experiences shared by older couples, which may influence variability in their subjective age.

